# Enhancing concealed object detection in active THz security images with adaptation-YOLO

**DOI:** 10.1038/s41598-024-81054-1

**Published:** 2025-01-21

**Authors:** Aiguo Cheng, Shiyou Wu, Xiaodong Liu, Hangyu Lu

**Affiliations:** 1 Aerospace Information Research Institute, Chinese Academy of Sciences, Beijing, 100190 China; 2 Key Laboratory of Electromagnetic Radiation and Sensing Technology, Chinese Academy of Sciences, Beijing, 100190 China; 3 School of Electronic, Electrical and Communication Engineering, University of Chinese Academy of Sciences, Beijing, 100049 China

**Keywords:** Concealed object detection, Attention mechanism, Adaptive convolution, Active terahertz (THz) security image, Electrical and electronic engineering, Computer science

## Abstract

The terahertz (THz) security scanner offers advantages such as non-contact inspection and the ability to detect various types of dangerous goods, playing an important role in preventing terrorist attacks. We aim to accurately and quickly detect concealed objects in THz security images. However, current object detection algorithms face many challenges when applied to THz images. The main reasons for the detection difficulty are that the concealed objects are small, the image resolution is low, and there is back-ground noise. Many methods often ignore the contextual dependency of the objects, hindering the effective capture of the object’s features. To address this task, this paper first proposes an adaptive context-aware attention network (ACAN), which models global contextual association features in both spatial and channel dimensions. By dynamically combining local features and their global relationships, contextual association information can be obtained from the input features, and enhanced attention features can be achieved through feature fusion to enable precise detection of concealed objects. Secondly, we improved the adaptive convolution and developed the dynamic adaptive convolution block (DACB). DACB can adaptively adjust convolution filter parameters and allocate the filters to the corresponding spatial regions, then filter the feature maps to suppress interference information. Finally, we integrated these two components to YOLOv8, resulting in Adaptation-YOLO. Through wide-ranging experiments on the active THz image dataset, the results demonstrate that the suggested method effectively improves the accuracy and efficiency of object detectors.

## Introduction

To effectively meet the security inspection requirements in crowded scenarios and prevent terrorist attacks^1–3^, there is an urgent need to design efficient and reliable security systems. Traditional security inspection facilities require manual assistance to complete the inspection, resulting in low efficiency. They can only detect dangerous items in backpacks and metal objects carried by individuals, and X-rays can also cause some radiation exposure to the human body^4^.

Terahertz (THz) waves lie between far-infrared and millimeter waves in the electromagnetic spectrum, with wavelengths ranging from approximately 1 mm to 100 μm. Electromagnetic waves in this frequency band possess both the penetrative ability of microwave and millimeter waves and the collimation of light waves, and they have extremely abundant spectral resources. Because THz waves are hard to detect with traditional electronic and optical techniques, this range is often referred to as the last uncharted territory of the electromagnetic spectrum^5^, known as the THz gap.

In the past two or three decades, THz radar imaging technology has made rapid advancements^6,7^, particularly in its excellent performance in the field of security inspection equipment: (1) Low Radiation: THz waves have relatively low radiation energy. When biological tissues are exposed to THz beams, they do not produce the photoionization effect of high-energy rays. Therefore, THz waves are suitable for security checks in densely populated public places. (2) Good Penetration: THz waves can penetrate foam, cardboard, and gypsum with low loss, effectively meeting the security inspection needs for objects in most scenarios. (3) High Resolution: Compared to the microwave band, the frequency band of THz waves is higher, resulting in shorter wavelengths. Combined with the excellent characteristics of wide bandwidth, THz waves can achieve higher imaging resolution, which helps improve the accuracy of object recognition. Therefore, THz imaging systems have the characteristics of safety, privacy protection, and rapid imaging. Based on these advantages, THz security screening systems have great potential and have begun to be applied in various security check scenarios.

Recent research results indicate that convolutional neural network^8^ (CNN) can effectively extract image features containing intricate background noise and aid in the recognition of concealed objects in various scenarios^9–11^. Thanks to the rapid enhancement of hardware computing power, deep learning has made significant progress. Especially, CNN-based object detection algorithms can extract and learn the feature information of images. By updating the detection model parameters through loss functions and backpropagation^8^, these algorithms can ultimately achieve the detection and recognition of various objects. Many detectors^12–20^ have demonstrated impressive detection speed and accuracy, achieving high detection performance on multiple benchmark datasets. Therefore, applying deep learning-based object detection algorithms in THz security screening systems has become a technological trend.

The classification of single-stage and two-stage detectors is founded on whether or not they use region proposals. Two-stage detectors first generate a set of candidate object anchor boxes, which are distributed across the input image at multiple aspect ratios, and then regress and classify each box. During the object detection process, the model predicts the offset and type of each bounding box, thereby achieving object detection. R-CNN^12^ is a typical two-stage detector. It first generates thousands of candidate regions using the selective search method, then extracts features using a pre-trained CNN, and finally determines whether each candidate region contains a specific object category. Fast R-CNN^13^ and Faster R-CNN^14^ are improved versions of R-CNN; both introduce a separate region proposal network (RPN) to generate potential regions, sharing the same convolutional feature extraction layers with the detection module. RetinaNet^15^ uses a feature pyramid network^16^ (FPN) to generate image features. FPN can capture rich semantic information at various scales, which aids in the detection of various sizes and aspect ratios. Additionally, by introducing focal loss, it addresses the class imbalance problem.

Single-stage detectors do not rely on RPN but directly extract the positions and features of objects from the image. These detectors typically use dense sampling or adaptive methods for object regression localization and classification. This makes the model simpler and more efficient, achieving faster inference speeds in object detection. YOLOv1^17^ divides the entire image into fixed-size grids and then predicts the position and class of objects within each grid cell. This allows for dense prediction across the entire image, improving detection efficiency and accuracy. YOLOv3^18^ improves and enhances performance and functionality by introducing three different scale detection layers. This multi-scale prediction mechanism helps in accurately detecting both small and large objects. YOLOv5 adopts an adaptive training strategy and a single-model multi-scale prediction strategy, using a lightweight network structure with fewer parameters and computational requirements. This achieves faster inference speeds and lower latency. Additionally, the SSD^19^ algorithm utilizes multi-level feature maps to detect objects of various sizes. By making predictions at different levels, SSD can effectively detect objects of different sizes and proportions, providing better adaptability. DSOD^20^ promotes information transfer and feature sharing by introducing dense connections between different levels, achieving multi-scale object prediction.

However, unlike natural images, raw THz security images have several drawbacks, such as insufficient texture information, background image noise, incomplete imaging of concealed objects, low contrast, and variable target shapes. Directly transferring these algorithms to the detection of concealed objects in THz security images does not achieve practical application results^21^. This is because identifying concealed objects at low resolution requires both coarse-grained positional features and fine-grained object-specific information. These algorithms struggle to effectively capture features in images containing complex noise and fail to accurately align the features of concealed small objects. Consequently, directly transplanting detection algorithms designed for natural images to THz images results in significant degradation in detection performance and fails to effectively detect and identify suspicious objects.

To solve these issues, we propose a fast automatic detection and recognition method for concealed objects in real-world THz security images. Firstly, we introduce a novel attention network called the adaptive context-aware attention network (ACAN). This attention network includes spatial and channel attention modules. Unlike the classic CBAM^22^, ACAN can adaptively model global context associations across both channel and spatial dimensions. In the spatial dimension, ACAN captures the feature of concealed objects based on a global context-aware attention mechanism. Briefly, it first computes a weight vector for all pixel values in the feature map using the softmax function. This weight vector is then applied to the input map to further adaptively generate a feature relationship dependency matrix between pixels. By modeling the global association between pixels through this dependency matrix, ACAN can better align the features of concealed targets. Finally, the feature relationship dependency matrix is applied to the input map to obtain the spatial attention map, which is then processed further. In the channel dimension, ACAN uses global average pooling (GAP) and convolution operations to simulate channel dependencies within each channel branch, thereby adaptively calibrating channel feature responses. This enhances the attention network’s sensitivity to channel feature information. Finally, the fusion of these two attention maps is achieved through matrix addition.

Additionally, to suppress background noise interference and further improve detection precision and recognition accuracy, we improved the adaptive convolution block^23^ (ACB) and developed the dynamic adaptive convolution block (DACB). DACB can adaptively adjust the convolution filter parameters based on the current feature map, generating adaptive convolution filters. It also generates dynamic guiding masks dependent on the input features and automatically determines the distribution of multiple filters. The resulting adaptive convolution filters are then used to filter the feature map, allowing the handling of variable spatial semantic distributions and generating enhanced features. This adaptive convolution can extract more discriminative features for detecting concealed objects while maintaining translation invariance for positions with similar features.

Finally, we integrated ACAN into YOLOv8 and replaced the convolution modules of neck and decoupled prediction head in YOLOv8 with DACB. This resulted in an improved version of YOLOv8, which we call Adaptation-YOLO.

The advancements presented in this paper are outlined as follows:

1. We introduce the ACAN for concealed object detection. ACAN includes spatial attention modules and channel attention modules. It can adaptively integrate high-level contextual information on the feature maps. ACAN adaptively models the global context dependencies of spatial pixels and channel feature responses, generating spatial and channel attention maps. Finally, these attention maps are fused through matrix addition to obtain enhanced attention feature representations. The enhanced attention features contain rich spatial intricacies and contextual semantic information, effectively facilitating the detection of concealed objects.
2. To handle the variable and complicated spatial information distribution, thereby further improving detection accuracy, we propose the DACB. DACB can adaptively adjust the parameters of convolution filters based on the current feature map, generating adaptive convolution filters. It also automatically creates a region-sharing pattern for the filters according to the characteristics of each input image. By using a guiding mask, it automatically allocates filters to the corresponding spatial dimension regions, filtering the respective feature map regions to obtain enhanced representations. Ultimately, this approach can better focus on concealed small objects, suppress interference from surrounding irrelevant information, and maintain translation invariance.
3. By integrating ACAN and DACB into the YOLOv8 detection framework, we developed an improved detection network called Adaptation-YOLO.
4. Extensive experiments on an active THz image dataset^24^ illustrate that the suggested method is efficacious. The detection efficiency of the improved network has been significantly enhanced, clearly surpassing previous methods.

## Related work

### Concealed object detection in THz security image

Traditional methods^25–29^ in THz object detection depend on hand-crafted features to describe objects. These features are limited to specific object types, thereby restricting the feature representation of objects and leading to ineffective capture of object features. For example, L.C. Ramac et al.^25^ proposed a method that combines morphological filters with wavelets to improve the recognition performance of concealed weapons in fused images. Otsu^26^ proposed an automatic threshold selection method to separate hidden weapons from other parts of the image. Dalal et al.^27^ used a histogram of oriented gradients (HOG) for object classification and detection. The Slamani Mapping Procedure^28^ (SMP) quantifies and decomposes fused images into multiple uniform regions. Another method^29^ proposed by Slamani et al. uses shape parameters to examine each region for identification.

Nevertheless, these methods exhibit inadequate detection performance, low robustness, high false alarm rates, and are not suitable for practical applications.

With the proposal of various new types of networks, deep learning-based detectors have achieved great success on multiple large-scale object detection datasets^30,31^, and corresponding methods^32–35^ for concealed object detection have been actively explored. Xiao et al. combined Faster R-CNN’s preprocessing and structural optimization to propose a fast detection framework called R-PCNN^32^, which effectively improves the efficiency and precision of object detection in human THz images. Ou et al. proposed a segmentation approach for active THz images^33^, which can accurately segment concealed objects in active THz images. Yang et al.‘s method consists of rough detection and detailed recognition^34^. However, since this method incorporates sparse low-rank decomposition and Faster R-CNN is a region-based algorithm, it inevitably increases additional computational load and model inference time. Cheng et al. based their work on the SSD algorithm^35^, introducing residual networks, CBAM, and focal loss, achieving good detection performance on passive THz security images.

However, these studies either used two-stage detection algorithms, resulting in slower detection speeds, or they could not effectively suppress background noise interference. Consequently, they struggled to align the features of concealed objects accurately to perceive the objects, leading to imprecise object localization. Therefore, detection performance can still be greatly enhanced.

### Attention mechanism in computer vision

Attention mechanisms^36–47^ have been proven to aid various computer vision tasks. They can help models selectively focus on important regions or features in an image by adjusting the weights of varied channels or spatial positions in the feature map. This augments the feature encoding of object areas and suppresses the interference of irrelevant features, thereby improving the model’s perception capabilities.

Self-attention mechanisms have been extensively explored across multiple applications. The work “Attention Is All You Need”^36^ first introduced self-attention to extract global dependencies in the input, applying it to machine translation. SENet^37^ focuses on channel dimension features, modeling interdependencies between channels through two steps: squeeze and excitation. ECA^38^, an improvement on the SE module, introduces a local attention mechanism by applying local convolution operations on each channel to compute inter-channel attention weights, effectively implementing a local cross-channel interaction strategy. CBAM^22^ enhances important features and suppresses unimportant ones by deriving channel and spatial attention maps, which assign weights to the respective dimensions, thereby improving the model’s representation capability. BAM^39^ places the module at each bottleneck of the model, inferring attention maps along independent channel and spatial paths, constructing hierarchical attention. GALA^40^ is a network designed to learn a complex combination of local saliency and global context modulation. AA^41^ enhances image representation by extending relative self-attention to 2D inputs without relying on fully convolutional network (FCN) pre-training. SK^42^ enhances the CNN’s ability to capture multi-scale information by dynamically selecting the most suitable convolution kernel, thereby improving the model’s expressiveness and flexibility. TA^43^ augments the feature encoding capability of CNNs by rotating the feature maps along different axes and applying attention mechanisms to capture multi-dimensional information. GSoP^44^ captures holistic statistical correlations across the entire deep CNN by introducing global second-order pooling. CA^45^ captures far-reaching dependencies and encodes precise positional information, enabling it to capture far-reaching dependencies and retain precise positional information in the spatial dimension. FcaNet^46^ views channel attention as a compression predicament and incorporates discrete cosine transform into channel attention, extending it to the frequency domain. DWAN^47^ effectively combines the numerical properties of low-frequency and high-frequency bands to obtain channel magnitudes, better measuring each channel’s importance and coordinating structural and spatial attention across different channels.

Compared to the aforementioned methods, we propose the ACAN that includes both spatial and channel dimensions. ACAN aims to use the attention mechanism to provide guidance on contextual feature information in images, thereby adaptively enhancing the features of concealed objects and suppressing irrelevant features.

### Convolution in object detection algorithms

The robust feature extraction proficiencies of CNNs have led to significant achievements in computer vision task. However, the current mainstream convolution operations are executed in a way that shares weights in the spatial domain, which means that higher-level feature information can only be effectively extracted by stacking multiple convolution layers. Additionally, since each convolution layer has a fixed receptive field size, using a specific feature map makes it difficult to effectively detect objects of different scales.

To effectively model spatial domain feature information, researchers have proposed several improved methods. Dilated convolution^48^ increases the effective size of kernel, permitting the network to encompass a larger receptive field and effectively gather object information without increasing the number of convolutional parameters. However, they have poor feature extraction capabilities for small objects. Jeon et al.^49^ introduced an active convolution unit (ACU), which yields non-fixed shapes because they can comprehend any structure through backpropagation in the training process. ACU uses learned offsets to enhance sampling positions in convolutions, and these offsets become static after training. Flattened convolution^50^ significantly reduces the computational cost of convolution by separating the kernel along the channel dimension and the two spatial dimensions. Deformable convolutional networks^51^ adapts the sampling locations of each convolution kernel through learning, allowing the convolution operation to adjust adaptively and thereby capture more image details and structural information. Involution^52^ is a convolutional kernel that is spatially parameter-independent and channel parameter-shared. Spatial parameter independence allows the convolutional kernel to adjust to diverse visual patterns at various spatial positions, while channel parameter sharing reduces inter-channel redundancy. R-FCN^53^ uses a region-based FCN to derive local features. It amplifies the output channels by a factor of $3 \times 3,$ then selects the corresponding sub-tensors from different channels to form $3 \times 3$ blocks. Sparse convolution^54^ performs convolution operations only on non-zero pixels in the input image, thereby achieving efficient computation of sparse data.

Unlike these methods, our proposed DACB first generates a set of filters through an adaptive filter generation network. Then, using guiding masks, it selectively allocates different convolution kernels to spatial regions with similar input features, and finally performs the convolution operations. In this way, DACB can handle features with complex and variable spatial information distribution.

## Method

**Fig. 1:**
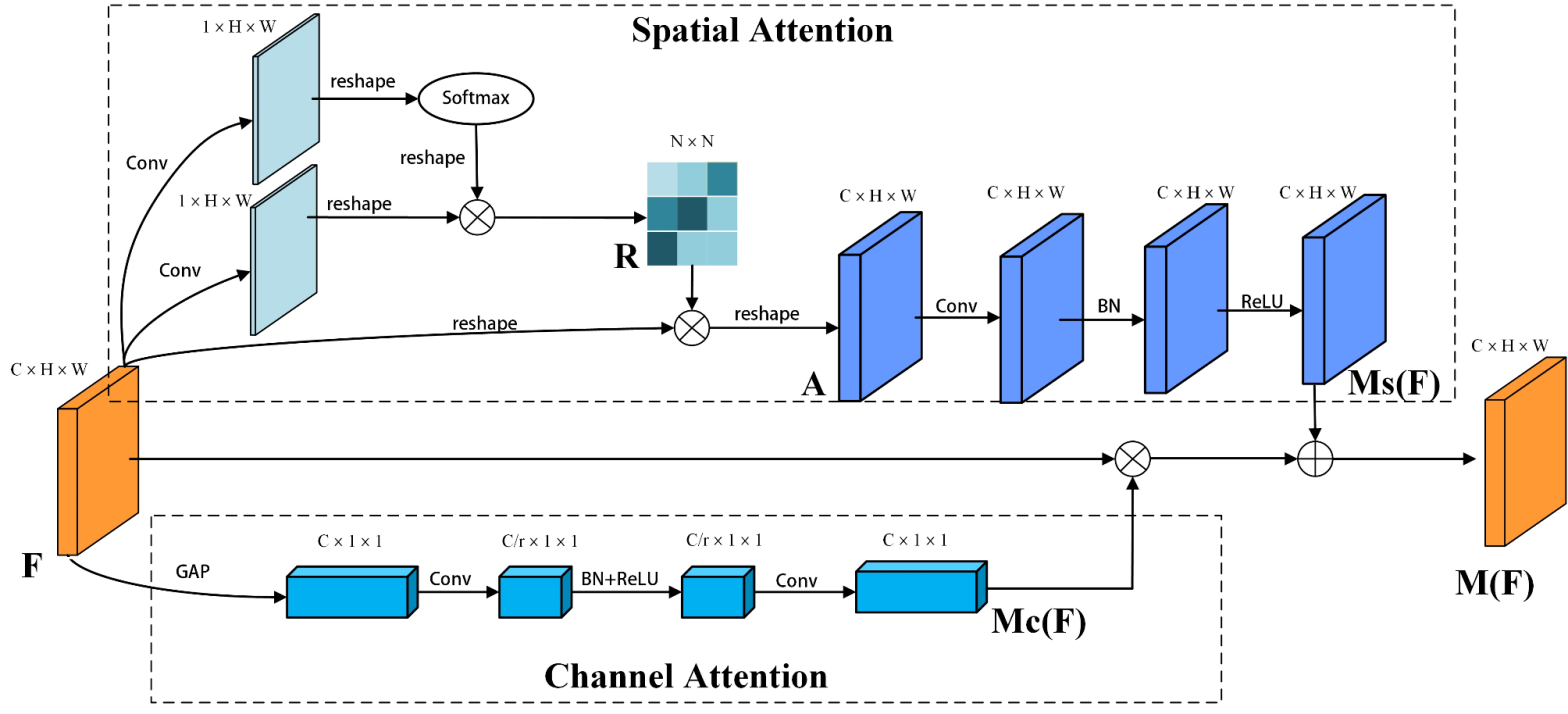
The detailed architecture of ACAN. Given a local feature map **F**, ACAN computes the corresponding attention maps through two independent attention branches (channel attention **Mc(F)** and spatial attention **Ms(F)**). Finally, the global context-aware attention feature map **M(F)** is obtained by element-wise summation of these attention maps.

In this study, we initially introduce the ACAN for THz concealed object recognition, as illustrated in Fig. 1. Due to the low resolution of THz security images, small hidden targets are easily affected by background noise. Therefore, we model the global contextual relationships of space and channels using this attention mechanism to help the detection network better capture the features of small concealed objects, thereby improving recognition precision. ACAN comprises spatial attention modules and channel attention modules, which can adaptively integrate contextual information of different scales on the various scale features produced by the backbone. It adaptively models the global context associations in both channel and spatial dimensions, generating attention feature maps in two dimensions. These maps are subsequently combined via matrix addition.

**Fig. 2:**
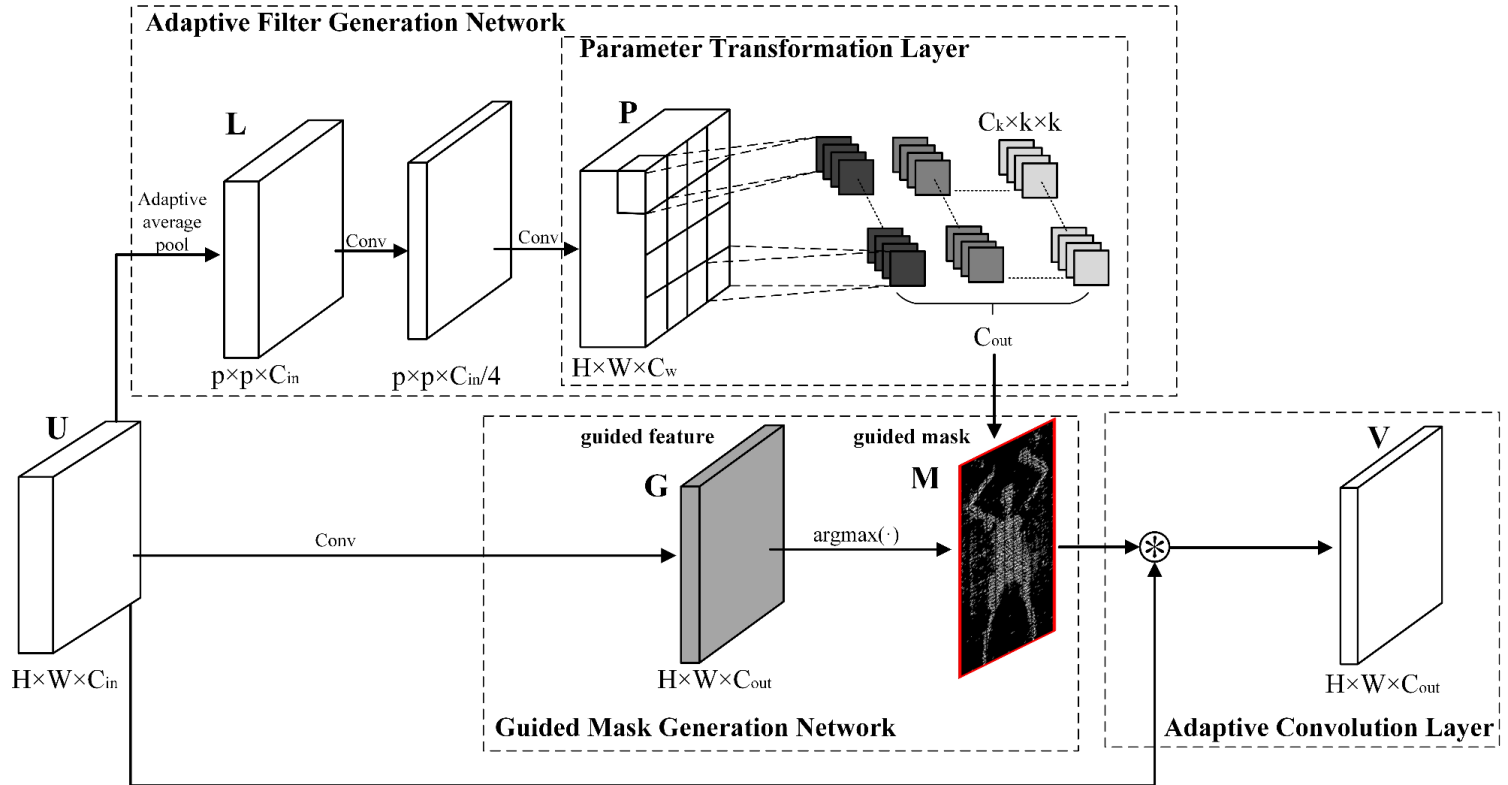
The detailed architecture of DACB. DACB is composed of three parts: AFGN, GMGN, and ACL. The AFGN also includes a PTL.

Secondly, to effectively address the issue of small concealed object features being easily overwhelmed during forward propagation in the network and to additionally enhance detection accuracy, we introduce the DACB, as illustrated in Fig. 2. DACB can adaptively adjust the convolution filter parameters based on the current feature map, generating adaptive convolution filters. It also generates dynamic guiding masks based on input features and automatically determines the distribution of multiple filters. The resulting filters are then used to filter the feature map, allowing the handling of variable spatial semantic distributions and generating enhanced features.

**Fig. 3:**
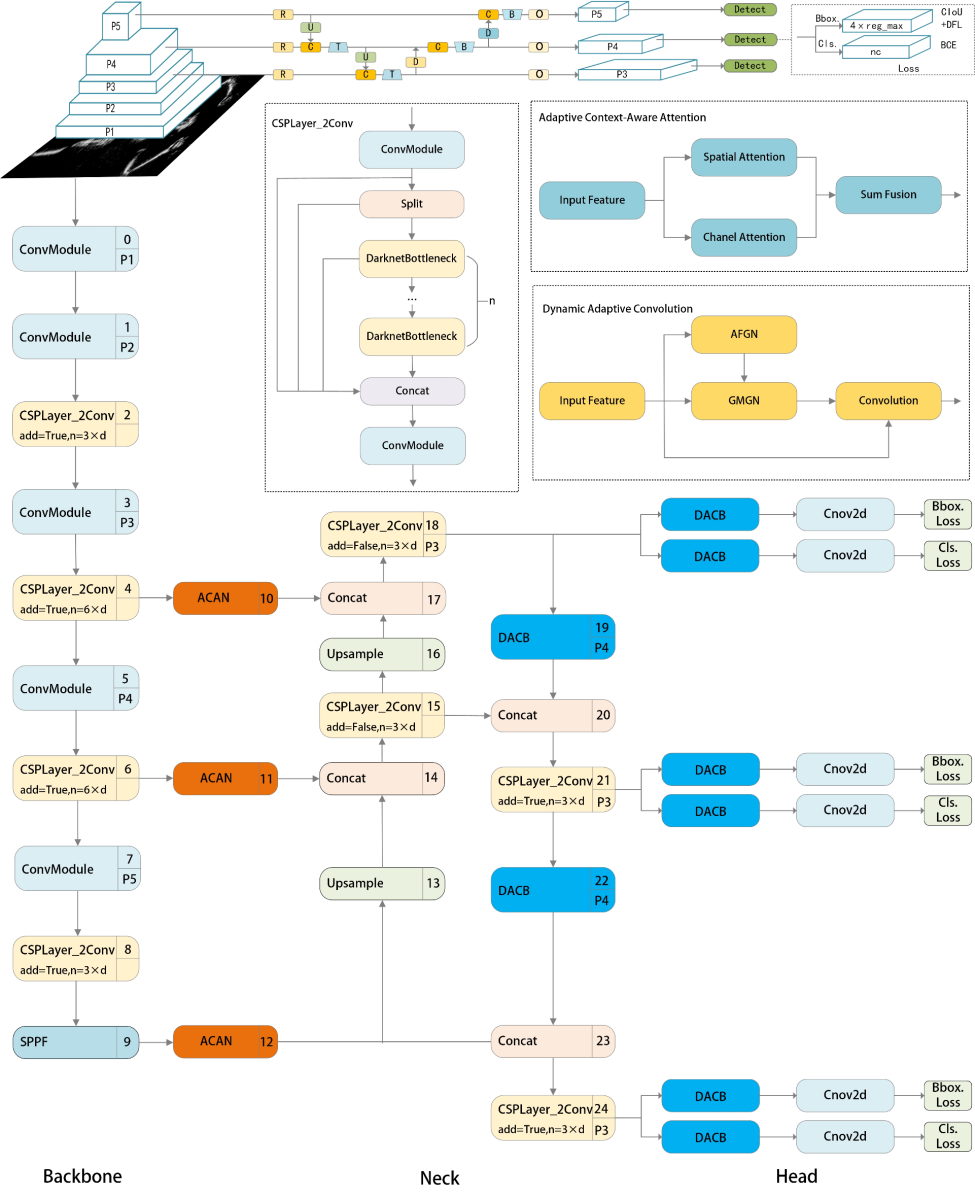
Overall Architecture of Adaptation-YOLO. The orange and blue modules are our proposed ACAN and DACB, respectively.

Therefore, we finally integrated ACAN and DACB into the YOLOv8 detection framework, resulting in the final improved YOLOv8 detection framework, namely Adaptation-YOLO, as illustrated in Fig. 3.

### Ethical statement

This study conforms to the ethical guidelines of the Declaration of Helsinki revised in 2013. The study was approved by the ethics committee of the Aerospace Information Research Institute, Chinese Academy of Sciences. All experiments were performed by relevant guidelines and regulations. We confirmed that informed consent had been obtained from all subjects. All images were deidentified before inclusion in this study.

### Adaptive context-aware attention network

Since the objects in active THz security images are very small and vary in shape and size, this can ultimately lead to an increase in false alarm rates. Therefore, it’s necessary to simultaneously utilize detailed spatial and channel dimension global contextual semantic information to accurately locate and classify small objects in THz images. Additionally, although some methods^55–57^ have proposed encoder-decoder frameworks to integrate basic and advanced semantic features to enhance spatial details, or have used recurrent neural networks^58,59^ (RNNs) to model long-range dependencies, which help in capturing objects of different scales and sizes and somewhat improves the deficiencies of traditional CNN, they fail to leverage multi-scale global contextual information and ignore the spatial domain relationships of the objects.

To effectively tackle this problem, we propose the ACAN, as shown in Fig. 1. ACAN establishes a global context relationship-aware attention mechanism model within the input features. This model can build semantic associations and derive guidance from attention maps rich in semantic information. Subsequently, it adaptively integrates similar features of any scale from a global perspective, enhancing them by perceiving and integrating globally context-weighted features, thereby merging related feature points adaptively. This efficient and multidimensional contextual feature information fusion enhances feature representation, suppresses information irrelevant to the object features, and effectively models contextual dependencies. Consequently, it improves pixel-level recognition rates.

#### Spatial attention

Specifically, our proposed spatial attention sub-module is illustrated in the upper part of Fig. 1. Suppose the input regional feature representation is ${\mathbf{F}} \in {{\mathbb{R}}^{C \times H \times W}}$. First, two new feature maps of size ${{\mathbb{R}}^{1 \times H \times W}}$ are obtained through two $1 \times 1$ convolution layers, respectively. Then, we reshape them to ${{\mathbb{R}}^{1 \times N}}$, where $N=H \times W$. Next, the first output feature is applied to the softmax function to compute the weight vector, which carries the spatial global context attention information. This weight vector is then multiplied by the second output feature through matrix multiplication, adaptively generating a feature relationship dependency matrix $\mathbf{R} \in {{\mathbb{R}}^{N \times N}}$ between pixels. Simultaneously, the initial feature map ${\mathbf{F}} \in {{\mathbb{R}}^{C \times H \times W}}$ is reshaped into ${{\mathbb{R}}^{{\text{C}} \times N}}$, which is then multiplied by the feature relationship dependency matrix $\mathbf{R} \in {{\mathbb{R}}^{N \times N}}$using matrix multiplication and further resized to obtain the final spatial context-related attention feature map $\mathbf{A} \in {{\mathbb{R}}^{C \times H \times W}}$.

The process can be described as follows:

1 $${\mathbf{A}}={\mathbf{F}} \otimes {{\mathbf{W}}_{\mathbf{1}}}\sum\nolimits_{{j=1}}^{N} {\frac{{\exp ({{\mathbf{W}}_{\mathbf{0}}}{{\mathbf{F}}_{\varvec{j}}})}}{{\sum\nolimits_{{i=1}}^{N} {\exp ({{\mathbf{W}}_{\mathbf{0}}}{{\mathbf{F}}_i})} }}} {{\mathbf{F}}_{\varvec{j}}}$$

Here, ${{\mathbf{W}}_{\mathbf{0}}}$, ${{\mathbf{W}}_{\mathbf{1}}}$ represent the weights of the two convolution layers in the spatial attention module, respectively. The spatial context-related attention feature map${\mathbf{A}}$includes the relational dependencies between the *i*-th position and the *j*-th position. The symbol$\otimes$denotes the matrix multiplication operation.

Further, we sequentially input the spatial context-related attention feature map ${\mathbf{A}}$ into a $1 \times 1$ convolution layer and a batch normalization (BN) layer for further sampling to derive high-level features and adjust the scale of the spatial branch output. Finally, the ReLU is applied to produce the final output spatial attention map ${\mathbf{M}_\mathbf{S}}(\mathbf{F}) \in {{\mathbb{R}}^{C \times H \times W}}$.The procedure can be represented as follows:

2 $${{\mathbf{M}}_\mathbf{s}}({\mathbf{F}})=\operatorname{Re} \text{L}\text{U}(BN(Conv(({\mathbf{A}}))))$$

Here, ${{\mathbf{M}}_\mathbf{s}}$ represents the spatial attention feature map obtained after applying spatial attention to ${\mathbf{F}} \in {{\mathbb{R}}^{C \times H \times W}}.$

#### Channel attention

The channel attention sub-module is illustrated in the lower part of Fig. 1. Since each high-level feature map extracted by the backbone contains specific channel feature responses and the semantic responses between any two channels are related, we utilize the feature dependencies between different channels in the channel branch to model the global context-related information features of the channels. Suppose the input regional feature representation is ${\mathbf{F}} \in {{\mathbb{R}}^{C \times H \times W}}$. To aggregate contextual information of each channel’s feature, we perform global average pooling (GAP) on the map ${\mathbf{F}} \in {{\mathbb{R}}^{C \times H \times W}}$, generating a channel feature relationship dependency vector of size ${{\mathbb{R}}^{C \times 1 \times 1}}$. This vector encapsulates the global context-related information within each channel. To obtain the attention for each channel from the channel feature relationship dependency vector, we input the channel feature relationship dependency vector into a $1 \times 1$ convolution layer to downsample the channel dimension, obtaining a feature map of size ${{\mathbb{R}}^{C/r \times 1 \times 1}}$, where *r* is the compression ratio. This is followed by a BN to adjust the scale of the channel context-related attention map output, then the ReLU activation function is applied. Finally, it is input into a $1 \times 1$ convolution layer to restore the channel contextual attention map to ${{\mathbb{R}}^{C \times 1 \times 1}}$, resulting in the final channel attention feature map ${\mathbf{M}_\mathbf{c}}(\mathbf{F}) \in {{\mathbb{R}}^{C \times 1 \times 1}}$. This map is then expanded to the dimensions of ${{\mathbb{R}}^{C \times H \times W}}$ and multiplied by the original map ${\mathbf{F}} \in {{\mathbb{R}}^{C \times H \times W}}$ using matrix multiplication. This process can be represented as:

3 $$\begin{gathered} {{\mathbf{M}}_{\mathbf{c}}}({\mathbf{F}})={\mathbf{F}} \otimes Conv(\operatorname{Re} \text{L}\text{U}(BN(Conv(AvgPool({\mathbf{F}})))) \\ ={\mathbf{F}} \otimes {{\mathbf{W}}_3}(\operatorname{Re} \text{L}\text{U}(BN(({{\mathbf{W}}_2}AvgPool({\mathbf{F}}))))) \\ \end{gathered}$$

Here, ${{\mathbf{M}}_{\mathbf{c}}}$ represents the channel attention feature map obtained after applying channel attention to the ${\mathbf{F}} \in {{\mathbb{R}}^{C \times H \times W}}$, ${{\mathbf{W}}_2}$ and ${{\mathbf{W}}_3}$ represent the weights of the two convolution layers in the channel attention module, respectively.

Further, after obtaining ${{\mathbf{M}}_{\mathbf{c}}}({\mathbf{F}})$ and ${\mathbf{M}_\mathbf{s}}({\mathbf{F}})$ from the two attention pipelines, we merge them to get the final enhanced attention feature map ${\mathbf{M}}({\mathbf{F}})$. Among various combination methods such as element-wise addition, element-wise multiplication, and maxi-mum operation, we choose element-wise addition to achieve efficient gradient flow. This can be represented as:

4 $${\mathbf{M}}({\mathbf{F}})={\mathbf{M}_\mathbf{s}}({\mathbf{F}}) \oplus {\mathbf{M}_\mathbf{c}}({\mathbf{F}})$$

### Dynamic adaptive convolution block

Owing to the low resolution of active THz security images, the presence of significant background noise, and the small size of concealed objects, detecting and recognizing these objects pose difficulties and challenges, often leading to an increased false positive rate. Traditional CNN shallow networks can only extract simple features from images, and the details of concealed objects are often obscured by background noise. During forward propagation in the network, the relevant feature information of small concealed objects may be overshadowed, reducing the recognition accuracy of these concealed objects.

Moreover, the limited receptive field of conventional convolutions can only capture semantic relationships within local regions of the image. The weight-sharing mechanism restricts standard convolution’s ability to model semantic variations, leading to inconsistent predictions in concealed object areas. Continuous downsampling also leads to the degradation of spatial details, ultimately increasing the false positive rate. Although adaptive convolution^23^ enhances the response to small concealed objects and reduces background noise interference, significantly improving upon conventional convolution, it only dynamically adjusts the filter parameters. It does not account for feature differences across various spatial regions, which are crucial for modeling semantic variations in images and for effective feature learning in detection networks. Inspired by dynamic region-aware convolution^60^, we introduced a dynamic feature mask mechanism, effectively improving this issue and further enhancing detection accuracy. We finally propose the DACB, whose structure is illustrated in Fig. 2.

Since traditional convolution filter parameters are usually fixed and can only be updated through backpropagation during training, DACB adaptively adjusts convolution filter parameters based on the current feature map, generating adaptive convolution filters that selectively extract features better suited to the current scene. Additionally, DACB adaptively generates dynamic guiding masks based on the characteristics of the input image, dividing the spatial dimension into multiple regions. These guiding masks then automatically allocate the filters to the corresponding spatial regions, filtering the respective feature map areas to generate enhanced features. The enhanced features processed by DACB can more effectively focus on concealed objects, reduce the interference of background information around the objects, maintain translation invariance, amplify the response of object areas, and handle complex and variable spatial information distributions. This enhancement improves feature representation in complex scenes, effectively increasing detection accuracy.

Specifically, we have innovatively introduced a dynamic feature mask mechanism built upon the original method^23^. Thus, DACB consists of an adaptive filter generation network (AFGN), an adaptive convolution layer (ACL), and a guiding mask generation network (GMGN).

#### Adaptive filter generation network

Assuming the local feature map input to the adaptive filter generation network is $\mathbf{U} \in {{\mathbb{R}}^{H \times W \times {C_{in}}}}$, it is first downsampled through an adaptive average pooling (AAP) layer to obtain $\mathbf{L} \in {{\mathbb{R}}^{{C_{in}} \times p \times p}}$. Then, this feature map is sequentially fed into two regular convolution layers to obtain filter parameters $\mathbf{P} \in {{\mathbb{R}}^{H \times W \times {C_w}}}$, with the number of channels being ${C_w}={C_{out}} \times {C_k} \times {k^2}/{p^2}$. After reorganizing $\mathbf{P}$, an array of learnable filters $\mathbf{W} \in {{\mathbb{R}}^{{C_{out}} \times {C_k} \times k \times k}}$ is obtained, where ${C_k}$ represents the amount of convolution kernels in each set of filters, and the convolution kernel size is $k \times k$.

The computation of AAP can be described as follows:

5 $${\mathbf{L}_{c,h,w}}=\frac{1}{N}\sum\limits_{{(i,j) \in {\Omega _{h,w}}}} {{\mathbf{U}_{c,i,j}}}$$

Where $$c \in \left\{ {1,2,\ldots,{C_{in}}} \right\}$$, $$h \in \left\{ {1,\ldots,p} \right\}$$, $$w \in \left\{ {1,\ldots,p} \right\}$$, $$\Omega _{{h,w}} = \left\{ {(i,j)\left| {{\text{ i}} \in } \right.,} \right.$$$$\left\{ {\left( {h - 1} \right)} \right.$$$$\times \left\lceil {H/p} \right\rceil , \ldots ,$$$$\left. {h \times \left\lceil {H/p} \right\rceil } \right\}$$$$j \in \left\{ {\left( {w - 1} \right)} \right.$$$$\times \left\lceil {H/p} \right\rceil , \ldots ,$$$$\left. {w \times \left\lceil {H/p} \right\rceil } \right\}$$, $$\left\lceil \cdot \right\rceil$$ represents the ceiling function, and *N* is the number of grids contained in region $$\Omega$$. By aggregating the information of region $$\Omega$$ into individual grids, the low-dimensional feature map $$\mathbf{U}$$, which contains rich object detail information, is embedded into the high-dimensional feature map $$\mathbf{L}$$ that contains higher-level semantic information. which better facilitates the accurate recognition of concealed objects.

PTL is a part of AFGN, as shown in Fig. 2. Its function is to reorganize the learned filters based on the regional feature information of different grids in feature map $\mathbf{L}$, enhancing the precise perception of local features by the filters. Unlike image segmentation, the detection of concealed small objects requires more focus on local details. Therefore, PTL generates the convolution kernel parameters for each collection of filters according to the same grid region of different channels in feature map $\mathbf{L}$ and constructs the filter parameters based on the corresponding grid. If there is a concealed object in a grid, the filters related to that grid region can detect the object and react, thereby enhancing the location perception capability of the filters. Specifically, assuming the obtained filter parameters are $\mathbf{P} \in {{\mathbb{R}}^{{C_w} \times p \times p}}$, PTL splits the parameters $\mathbf{P}$ with a channel dimension of ${C_w}$ into multiple independent channel bundles, with a total of $\left\lfloor {{C_w}/{k^2}} \right\rfloor$ bundles, where $\left\lfloor \cdot \right\rfloor$ represents the floor function. Each channel bundle contains ${k^2}$ adjacent channels. The filter parameters of a channel bundle are divided into a set of ${C_k}$ 2D convolution kernels with a dimension of $k \times k$. These convolution kernels form filters with a dimension of ${{\mathbb{R}}^{{C_k} \times k \times k}}$. Therefore, all the filters can ultimately be represented as $\mathbf{W} \in {{\mathbb{R}}^{{C_{out}} \times {C_k} \times k \times k}}$.

6 $${\mathbf{W}_{i,j,h,w}}={\mathbf{P}_{{C_{wi}},\left\lfloor {mod({k_i},{p^2})/p} \right\rfloor +1,mod({k_i},p)+1}}$$

where $i \in \left\{ {1,2,\ldots,{C_{out}}} \right\}$, $j \in \left\{ {1,2,\ldots,{C_k}} \right\}$, $h,w \in \left\{ {1,2, \ldots,k} \right\}$, ${C_{wi}}=\left\lfloor {{{{k_i}} \mathord{\left/ {\vphantom {{{k_i}} {{p^2}}}} \right. \kern-0pt} {{p^2}}}} \right\rfloor \cdot {k^2}+(x - 1) \cdot k+y,$ ${k_i}={C_k} \cdot (i - 1)+j - 1$, $mod()$ is the modulo operation.

#### Guiding mask generation network

After constructing the filter parameters, GMGN is responsible for allocating these filters. GMGN automatically determines the regional allocation of the filters through the generated dynamic guiding mask. This allows the filters to be adaptively allocated to the corresponding regions as the distribution of input feature space information changes, thereby aiding in modeling image semantic variations. Specifically, we input the local feature $\mathbf{U} \in {{\mathbb{R}}^{H \times W \times {C_{in}}}}$ into a regular convolution layer to generate guiding features with ${C_{out}}$ channels, and use $\mathbf{G} \in {{\mathbb{R}}^{H \times W \times {C_{out}}}}$ to represent the guiding features. Furthermore, we apply the $argmax( \cdot )$ function to the guiding features to generate the guiding mask, represented as $\mathbf{M} \in {{\mathbb{R}}^{H \times W}}$. The input features are then spatially partitioned into regions based on the guiding mask. Therefore, for different spatial positions $(h,w)$, we have:

7 $${\mathbf{M}_{h,w}}=argmax(\mathbf{G}_{{h,w}}^{0},\mathbf{G}_{{h,w}}^{1}, \cdot \cdot \cdot ,\mathbf{G}_{{h,w}}^{{{C_{out}}-1}})$$

where $argmax( \cdot )$represents the index of the maximum value in each channel of the output guiding feature map, ${\mathbf{G}_{h,w}}$ denotes the guiding feature vector at position $(h,w)$, and ${C_{out}}$ represents the number of elements in the vector. Therefore, the values in the guiding mask range from 0 to ${C_{out}} - 1$, indicating the index of the filter that should be used at the corresponding position.

#### Adaptive convolution layer

Finally, we perform the convolution operation between the input feature $\mathbf{U} \in {{\mathbb{R}}^{H \times W \times {C_{in}}}}$ and the allocated filter $\mathbf{W} \in {{\mathbb{R}}^{{C_{out}} \times {C_k} \times k \times k}}$ through ACL, resulting in the final output $\mathbf{V} \in {{\mathbb{R}}^{H \times W \times {C_{out}}}}$. This process is a standard convolution operation and can be represented as follows:

8 $${\mathbf{V}_i}=\sum\limits_{{i=1}}^{{{C_k}}} {{\mathbf{U}_{{C_{ij}}}} * {\mathbf{W}_{i,j}}}$$

where ${\mathbf{V}_i}$ represents the *i*-th channel of $\mathbf{V},$ ${\mathbf{U}_{Cij}}$ represents the ${C_{ij}}$-th channel of $\mathbf{U},$ ${C_{ij}}={C_{in}}/g \cdot \left\lfloor {i \cdot g/{C_{out}}} \right\rfloor +j,$ and ${\mathbf{W}_{ij}}$ represents the *j*-th convolution kernel of the *i*-th filter, as defined in (6).

### Adaptation-YOLO

YOLOv8 is a brand-new state-of-the-art (SOTA) model. The YOLOv8 framework mainly consists of three components: the backbone feature extraction network, the neck network (feature fusion network), and the head network (decoupled prediction head). The backbone and neck parts are inspired by the design philosophy of YOLOv7-ELAN and inherit the main structure of YOLOv5. It replaces YOLOv5’s C3 structure with the C2f structure, which provides richer gradient flow, more skip connections, and additional split operations. At the same time, two convolutional layers from the neck module have been removed. The head has been replaced with the modern decoupled head structure, which separates the classification and detection heads. Additionally, it shifts from an anchor-based design to an anchor-free one. The number of channels has also been adjusted for different scale models, representing meticulous structural fine-tuning that significantly improves performance. For loss calculation, YOLOv8 adopts the TaskAlignedAssigner strategy for positive sample assignment and introduces Distribution Focal Loss.

Although the different scale representations generated by the YOLOv8 feature extraction network contain rich details, they include both beneficial feature information for detection and recognition, as well as potentially misleading interference information. To effectively address this issue, we integrate the proposed ACAN into the YOLOv8 backbone, as illustrated in Fig. 3. We connect our ACAN to three different scale feature layers of the YOLOv8 backbone to create enhanced attention representations at different scales. In this way, we not only aggregate contextual feature information to better identify small concealed objects but also improve the fine-grained feature extraction information, aiding the detection network in achieving more accurate semantic predictions.

Additionally, since the shallow neurons of traditional CNN can only extract local features with weak semantic information, they cannot naturally distinguish concealed objects from surrounding interference.

In the complex and variable scenes of THz images, background noise is more likely to produce stronger feature responses than small concealed objects. This results in the information of small concealed objects being severely affected by background noise and other irrelevant features. Therefore, we replaced the convolution modules in the neck module and the decoupled prediction head of YOLOv8 with DACB. The final result is achieved through Adaptation-YOLO, as shown in Fig. 3.

## Experiments

### Dataset

We use the active THz image dataset^24^ for experiments to assess the suggested method. This dataset contains 11 types of various objects with diverse shapes and sizes. These objects include items such as guns, scissors, ceramic knives, cell phones, and leather wallets, as shown in Table 1. Ten volunteers participated equally in the experiments, each carrying 0 to 3 objects hidden at different positions on their bodies. Images were taken from both the front and back of each volunteer during each imaging session, with each image sized at $335 \times 880$ pixels. There is an aggregate of 3157 images in this dataset, and we separated them into a dataset (2526 images) for training and a dataset (631 images) for test.

**Table 1:** The categories and quantities of objects included in the dataset.

Category	GA	KK	SS	MD	CK	WB	KC	CP	CL	LW	HM
Object	Gun	Kitchen knife	Scissors	Metal dagger	Ceramic knife	Water bottle	Key chain	Cell phone	Cigarette lighter	Leather wallet	Human
Quantity	116	100	96	64	129	107	78	129	163	78	3157

### Implementation details

We use YOLOv8 as the base framework and integrate the proposed ACAN and DACB into this framework. The improved detection framework is implemented using Pytorch^61^. The hardware facilities for our experiments include an Intel Core i5-10400 F CPU and 4 GeForce RTX 3090Ti GPUs. All models are trained on this hardware setup, with the operating environment being Ubuntu 20.04, CUDA 10.2, and Python 3.9. Each raw image is adjusted to $960 \times 640$ before being input to the detection framework. We use SGD optimizer to adjust model parameters, where the momentum parameter is set to 0.8 and the weight decay parameter equals to 0.0005. Our training process consists of 300 epochs, with a total batch size of 64, allocating 16 images to each GPU. The primary learning rate is 0.003, and it is divided by 10 every 20 epochs. In the detection network, the feature maps from the P3, P4, and P5 layers are input into the ACAN to obtain enhanced attention features. Then, after feature fusion, the result is fed into the decoupled prediction module to obtain the prediction vector.

### Evaluation metrics

To measure the experimental results and detection performance, we employ the following performance measures. First, the intersection over union (IoU) is provided as a measure of overlap between the ground truth and the predicted box. Generally, when the IoU based on a given ground truth exceeds 0.5, we consider the predicted box correct. In other words, if the IoU is greater than the set threshold, it is identified as a true positive, whereas a false positive will have an IoU lower than this threshold. Average precision (AP) serves to assess the accuracy of detection for a specific class in a dataset, defined as the area under the precision-recall curve (P-R curve). Therefore, AP is derived as follows:

9 $$\text{A}\text{P}=\frac{1}{{11}}\sum\limits_{{r \in (0,0.1,\ldots,1)}} {{{\text{P}}_{intep}}(r)}$$

The mean average precision (mAP) value is calculated by averaging the AP values of all categories and is used to evaluate the average performance across all categories in the dataset.

10 $$\text{m}\text{A}\text{P}=\frac{1}{{\text{Q}}}\sum\limits_{{{\text{q}}=1}}^{{\text{Q}}} {\text{A}\text{P}(\text{q})}$$

Frames Per Second (FPS) refers to the number of images a model processes per second, which measures the speed of image processing.

### Experimental results and analyses

#### Ablation studies

The main innovations of this paper are the proposals of ACAN and DACB, which are integrated into the original YOLOv8 framework. The purpose of the ablation experiments is to separately study and analyze the effectiveness of the proposed ACAN and DACB. Since the proposed detection modules are based on the YOLOv8 detection framework, we use the YOLOv8 detection network as the baseline for all ablation studies. We focused on the improvements and enhancements that ACAN and DACB bring to YOLOv8. To verify that the proposed ACAN and DACB can enable the detection network to more accurately locate and classify concealed objects in THz images, we set up four different cases for comparison of detection performance: (1) Case1: the YOLOv8 detector; (2) Case2: integrating only DACB into the YOLOv8 framework; (3) Case3: integrating only ACAN into the YOLOv8 framework; (4) Case4: integrating both ACAN and DACB into the YOLOv8 framework to form Adaptation-YOLO.

**Table 2:** Comparison of detection performance for models obtained under four different experimental settings.

Network	AP											mAP	FPS
	CK	CL	CP	GA	HM	KC	KK	LW	MD	WB	SS		
Case1	51.5	28.4	69.4	67.3	99.9	59.2	79.4	**91.7**	30.5	78.7	**77.4**	66.7	17.61
Case2	**59.9**	32.6	69.8	66.4	99.9	**67.4**	**80.1**	90.3	41.9	69.0	76.6	68.5	17.61
Case3	56.3	32.4	70.5	70.4	99.9	63.9	76.5	86.4	48.5	**99.9**	73.8	70.8	17.54
Case4	56.3	**35.6**	**78.9**	**75.5**	99.9	61.6	75.8	79.5	**60.8**	84.5	74.8	**71.2**	17.46

Table 2 presents a comparison of the models under four different experimental settings during the testing process, including the AP values for each category, mAP values, and FPS. It can be seen that in Case2 and Case3, where DACB and ACAN are integrated respectively, the models show improvements in the detection performance of most single categories, corresponding to an increase in their AP values. Consequently, the overall mAP values of the models also increase. Specifically, compared to the baseline, the mAP of YOLOv8 integrated with DACB improved by 1.8%, while the mAP of YOLOv8 integrated with ACAN improved by 4.1%. In the final case, the mAP of our Adaptation-YOLO showed a 4.5% improvement over the baseline. The results indicate that the ACAN and DACB can effectively focus on and extract object features, diminish the representation of background noise, and achieve higher detection accuracy. Additionally, for the detection time per image, it can be seen from the table that, despite an increase in model parameters after integrating DACB and ACAN, the inference time does not significantly increase, with FPS consistently staying above 17. This is due to the model’s improved detection efficiency, enabling real-time prediction on a computer with average performance, thus meeting the security inspection needs of practical scenarios.

**Fig. 4:**
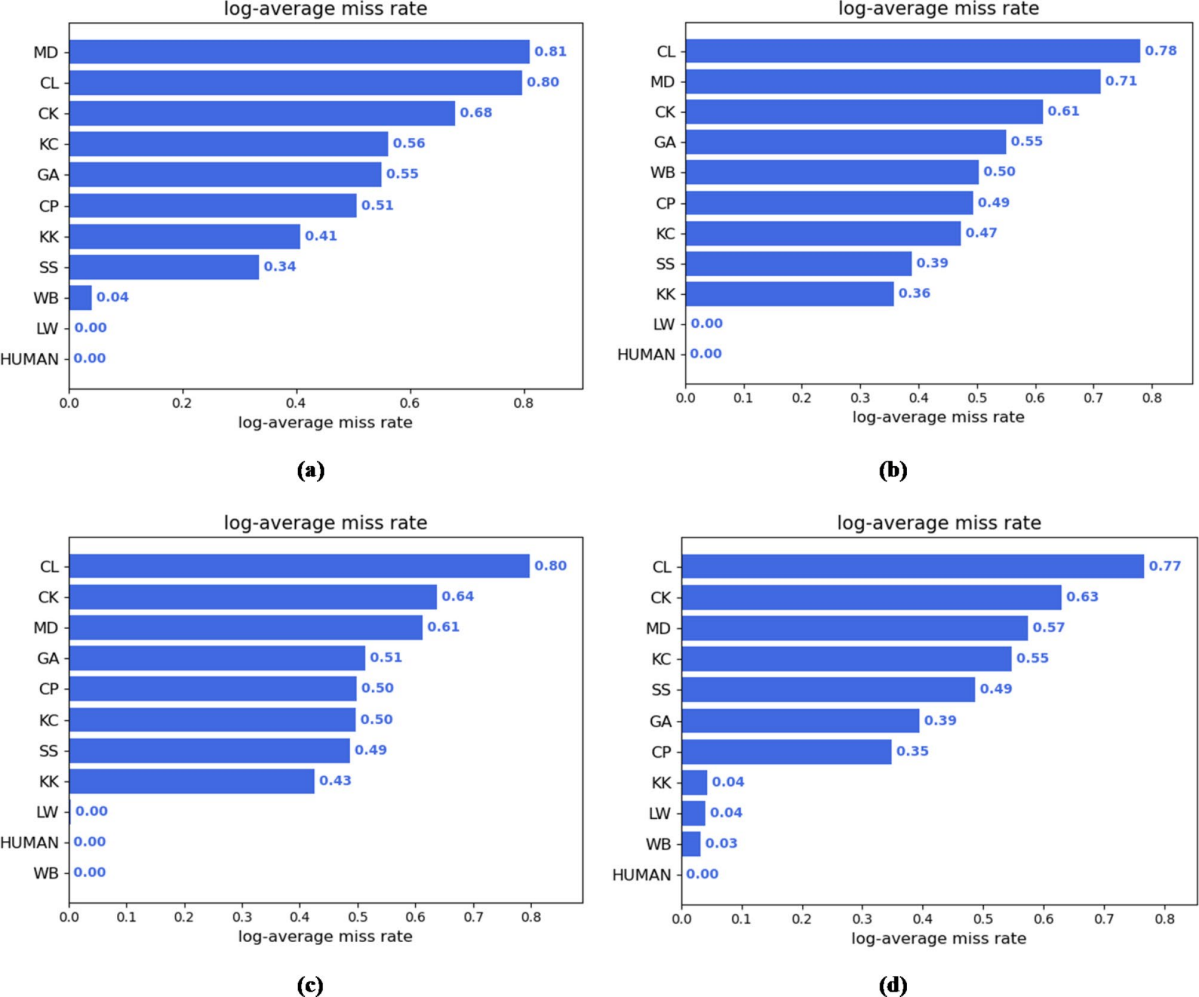
Comparison of log-average miss rates under four different experimental settings. (**a**) Case1. (**b**) Case2. (**c**) Case3. (**d**) Case4.

Figure 4 presents a comparison of the log-average miss rates for each category in the dataset across the four models. It can be seen that the baseline detector has a higher miss rate for object categories such as MD, CL, CK, KC, and GA. In Case2 and Case3, due to the integration of DACB and ACAN respectively, the miss rates for these categories decrease to varying degrees, demonstrating the effectiveness of both in reducing miss rates. In Case4, it can be observed that the miss rates for concealed objects across all categories continue to decrease, especially for categories such as GA, CP, KK, LW, WB and SS with miss rates below 50%. The reduction in the miss rate for GA is particularly noticeable, confirming the excellent detection performance of the Adaptation-YOLO.

**Fig. 5:**
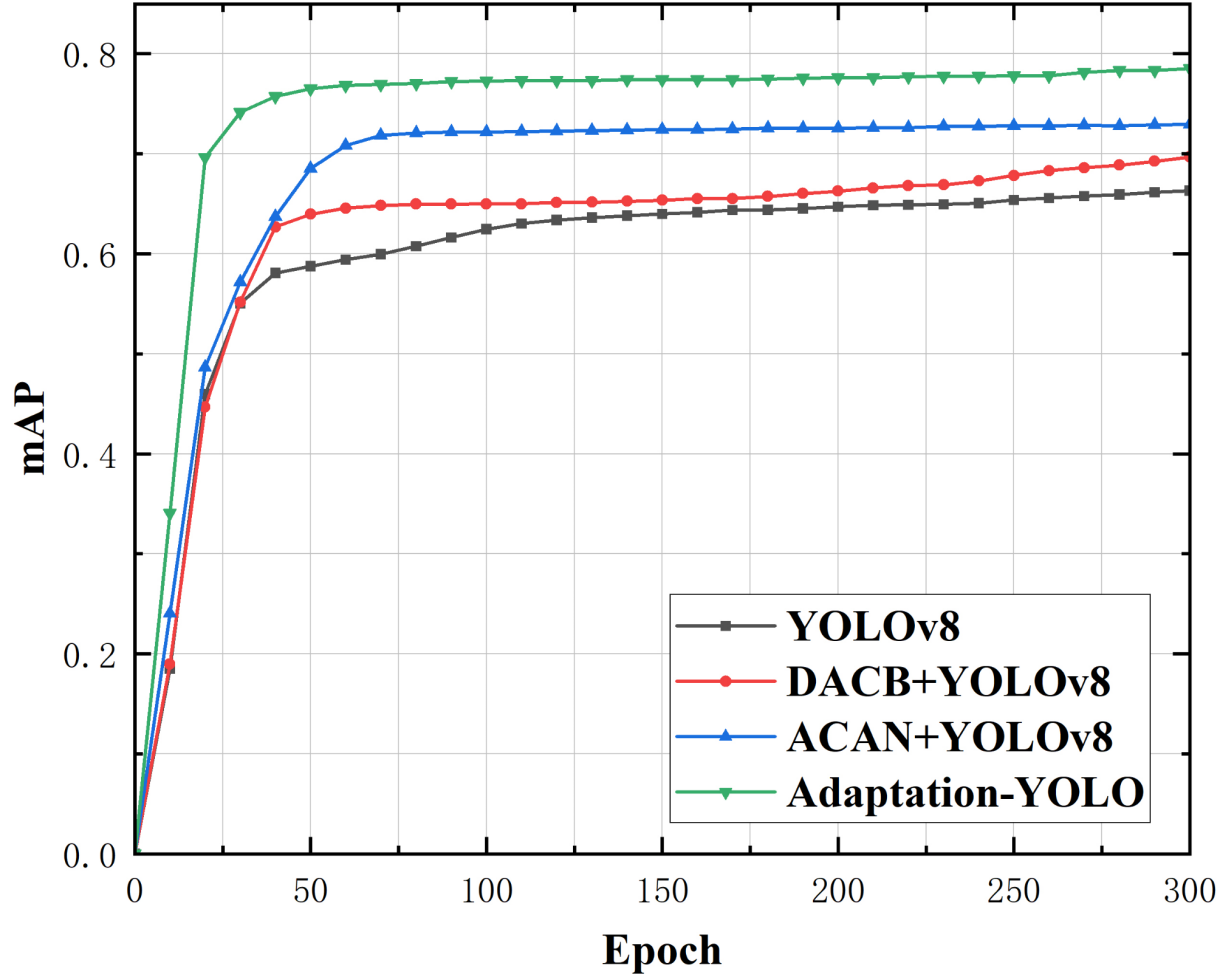
Comparison of mAP values of the four cases during the training process.

Additionally, since we emphasize the classification of concealed objects more during the detection process, the localization of objects becomes relatively less important. Therefore, we compared the changes in mAP values during the training process among the four cases, as shown in Fig. 5. It can be seen from the figure that the mAP value of YOLOv8 is lower compared to the other three cases. The YOLOv8 models integrated with DACB and ACAN show an increased rate of mAP improvement, surpassing the baseline method. The Adaptation-YOLO consistently achieves the highest mAP values during training, with the fastest increase rate, gradually stabilizing at a certain value. This indicates superior detection performance compared to the baseline method, demonstrating that the proposed method can learn the features of concealed objects more quickly and improve the model’s detection accuracy.

**Fig. 6:**
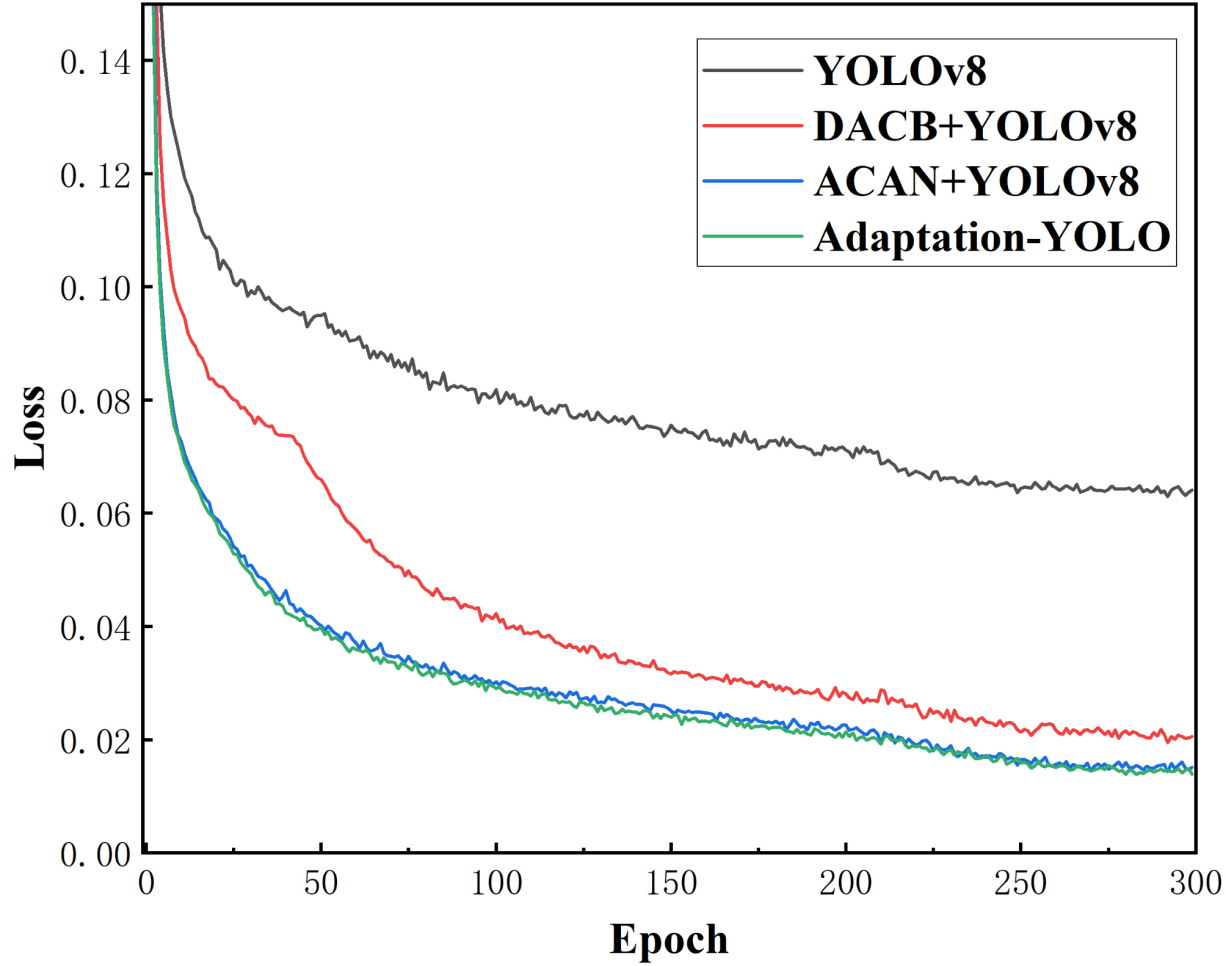
Comparison of loss values of the four models during the training process.

Finally, we compared the changes in loss values during the training process among the four models, as shown in Fig. 6. It is clearly visible that the baseline’s loss value decreases relatively slowly and remains high. In contrast, in the models integrated with DACB and ACAN, the loss values decrease significantly faster and reach lower levels. Particularly in the third case, the loss value drops rapidly after the start of training, and compared to the baseline, the loss function converges much faster during the training process. Its convergence rate is very close to that of Adaptation-YOLO, with the loss value curves nearly overlapping. This indicates that the proposed DACB and ACAN make the model easier to converge, and also suggests that the proposed methods enhance the efficiency and speed of learning object features, resulting in overall performance superior to the YOLOv8.

#### Comparison with state-of-the-art method

In this section, we objectively compare Adaptation-YOLO with state-of-the-art methods. First, we refer to experimental data reported in previous literature^11^, as shown in Table 3, which used the same dataset and evaluation methods as ours. Additionally, we quantitatively present the experimental results of our trained YOLOv8 and Adaptation-YOLO for detection performance across different categories and compare them with currently popular transformer-based methods, including ViT-FRCNN^62^ and YOLOS^63^, as shown in Table 4.

It can be seen that our method achieved excellent detection performance across all categories, obtaining the highest AP values in the CP, KC, MD, and HUMAN categories, effectively meeting the detection needs of practical applications. Particularly for the MD category, due to its metallic properties, small size, and similarity in shape to the CK category, previous methods struggled to achieve satisfactory detection results. However, our method reached an AP value of 60.8. The detection performance of ViT-FRCNN is slightly higher than that of YOLOv8; we believe ViT is more suitable for large-scale datasets due to its higher computational efficiency. YOLOS achieved improved detection performance, with an mAP value of 68.9. Ultimately, our method achieved an mAP of 71.2, the highest among all methods, representing an improvement of approximately 4.5% over baseline YOLOv8 and around 8% over YOLOv6, demonstrating the effectiveness of our proposed method in improving detection accuracy.

**Table 3:** Reported experimental results from the literature.

Network	AP											mAP
	CK	CL	CP	GA	HM	KC	KK	LW	MD	WB	SS	
Faster-RCNN	36.5	54.7	75.3	82.6	82.1	69.4	61.1	10.8	35.4	70.3	20.1	54.4
Grid-RCNN	38.8	57.2	80.5	87.5	87.5	74.5	64.7	11.3	36.7	74.2	22.2	57.7
DINO	43.5	64.3	90.6	97.8	94.4	83.4	72.9	13.7	41.4	82.8	24.9	64.5
Foucs-DETR	44.3	64.4	90.5	98.1	99.4	86.2	74.0	13.2	42.5	86.1	25.0	65.8
YOLOv5	20.1	42.9	85.6	99.5	99.5	94.2	61.8	13.2	44.7	81.7	22.9	60.6
YOLOX	27.9	53.0	85.4	96.6	96.6	87.0	62.7	11.4	37.4	94.6	34.5	62.5
YOLOv6	40.8	63.2	87.9	95.8	95.8	81.4	71.2	12.8	40.8	81.3	23.8	63.2
YOLOv7	40.3	55.9	88.3	99.5	99.5	89.9	65.6	14.3	40.3	97.5	37.4	66.2

**Table 4:** Comparison of experimental results between the proposed method and transformer methods.

Network	AP											mAP
	CK	CL	CP	GA	HM	KC	KK	LW	MD	WB	SS	
YOLOv8	51.5	28.4	69.4	67.3	99.9	59.2	**79.4**	**91.7**	30.5	78.7	**77.4**	66.7
ViT-FRCNN	59.9	34.4	78.3	**76.5**	99.9	53.2	70.6	88.9	45.7	90.5	42.5	67.3
YOLOS	**62.3**	**35.7**	75.7	71.5	99.9	58.9	65.4	88.9	42.3	**91.4**	66.2	68.9
ours	56.3	35.6	**78.9**	75.5	**99.9**	**61.6**	75.8	79.5	**60.8**	84.5	74.8	**71.2**

**Fig. 7:**
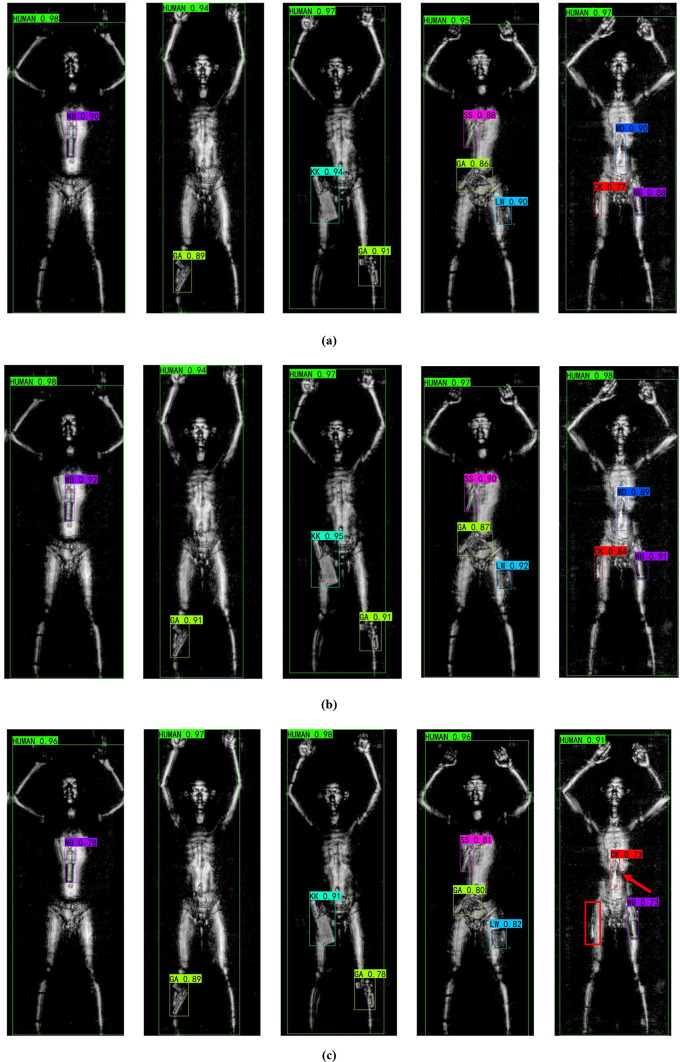

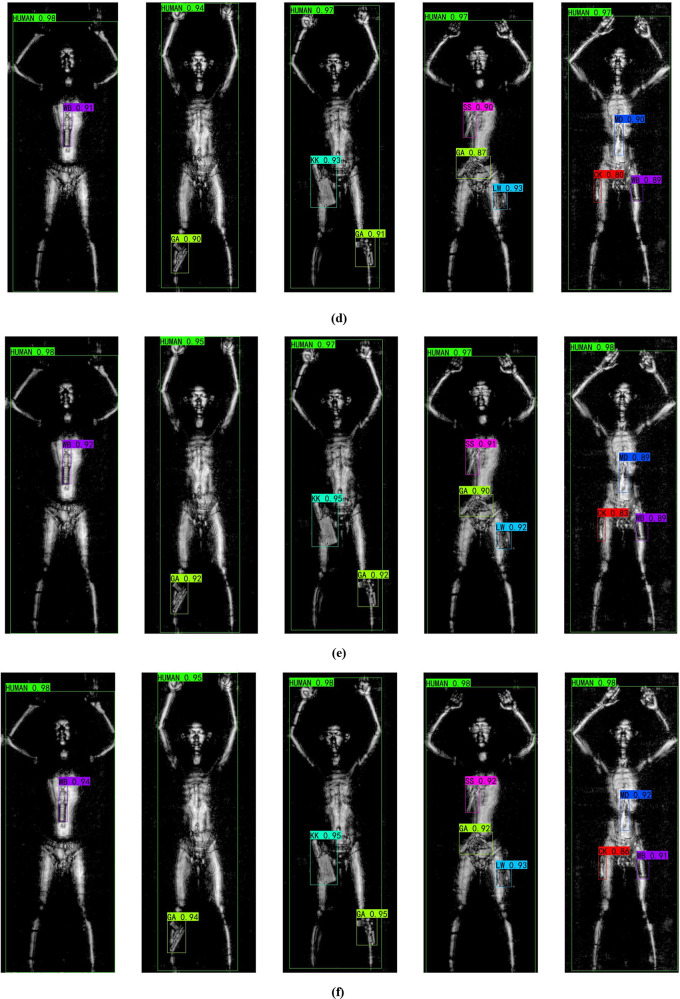
Comparison of the detection results between the proposed method and transformer methods on THz images containing different categories and quantities of objects. (**a**) ViT-FRCNN. (**b**) YOLOS. (**c**) Case1. (**d**) Case2. (**e**) Case3. (**f**) Case4.

In the final part of the experiment, we present sample detection results of the models trained under the four experimental settings described in section “Ablation studies” and compare them with the detection results of popular transformer methods. As shown in Fig. 7, each row corresponds to the detection results of a different model, covering single-object, two-object, and three-object scenarios. From the detection results, it is visually clear that the transformer-based methods did not produce any false positives or missed detections. YOLOS performed slightly better than ViT-FRCNN but still did not surpass our method. YOLOv8 showed lower detection accuracy and exhibited both misdetections and missed detections; for instance, in the fifth example, the model incorrectly identified an MD object as a CK object and failed to detect a CK object, these errors are marked with red arrows and boxes in the image. In case 2, the model can accurately classify concealed objects, but its detection accuracy is slightly lower. In case 3, the model’s detection accuracy is further improved, indicating that ACAN can effectively extract the contextual relationship features of concealed objects and achieve precise localization of objects. Finally, the proposed Adaptation-YOLO achieves the highest detection accuracy for various concealed objects, demonstrating that the integration of ACAN and DACB significantly enhances the model’s detection performance.

## Conclusion

In this paper, we propose the ACAN that can model contextual semantic association. By dynamically combining local features and their global relationships, enhanced contextual association feature attention information can be obtained from the extracted features. Secondly, we propose the DACB that can adaptively adjust convolution filter parameters based on the current feature map, generate adaptive convolution filters, dynamically create guiding masks based on input features, and automatically determine the distribution of multiple filters. Finally, the obtained adaptive convolution filters are used to filter the feature map. We integrated these two components into the YOLOv8 detection framework, resulting in the final improved detection network, Adaptation-YOLO. To assess our method, we carried out targeted experiments on the active THz image dataset. From the experimental results, it is evident that Adaptation-YOLO achieves higher detection accuracy compared to previous methods. Results from ablation studies demonstrate that our proposed ACAN and DACB effectively improve the model’s mAP values, aiding in the detection and recognition of concealed objects while effectively suppressing background noise. Considering that THz security imaging data from different inspection stations may have varying distributions in real-world scenarios, future research will explore introducing domain adaptation mechanisms and deep reinforcement learning to enhance the model’s generalization capabilities.

## Data Availability

This study uses publicly available datasets, which can be accessed through the GitHub repository at https://github.com/LingLIx/THz Dataset.
